# A Combination of Receptor-Based Pharmacophore Modeling & QM Techniques for Identification of Human Chymase Inhibitors

**DOI:** 10.1371/journal.pone.0063030

**Published:** 2013-04-26

**Authors:** Mahreen Arooj, Sugunadevi Sakkiah, Songmi Kim, Venkatesh Arulalapperumal, Keun Woo Lee

**Affiliations:** Division of Applied Life Science (BK21 Program), Systems and Synthetic Agrobiotech Center (SSAC), Plant Molecular Biology and Biotechnology Research Center (PMBBRC), Research Institute of Natural Science (RINS), Gyeongsang National University (GNU), Jinju, Republic of Korea; University of Delhi, India

## Abstract

Inhibition of chymase is likely to divulge therapeutic ways for the treatment of cardiovascular diseases, and fibrotic disorders. To find novel and potent chymase inhibitors and to provide a new idea for drug design, we used both ligand-based and structure-based methods to perform the virtual screening(VS) of commercially available databases. Different pharmacophore models generated from various crystal structures of enzyme may depict diverse inhibitor binding modes. Therefore, multiple pharmacophore-based approach is applied in this study. X-ray crystallographic data of chymase in complex with different inhibitors were used to generate four structure–based pharmacophore models. One ligand–based pharmacophore model was also developed from experimentally known inhibitors. After successful validation, all pharmacophore models were employed in database screening to retrieve hits with novel chemical scaffolds. Drug-like hit compounds were subjected to molecular docking using GOLD and AutoDock. Finally four structurally diverse compounds with high GOLD score and binding affinity for several crystal structures of chymase were selected as final hits. Identification of final hits by three different pharmacophore models necessitates the use of multiple pharmacophore-based approach in VS process. Quantum mechanical calculation is also conducted for analysis of electrostatic characteristics of compounds which illustrates their significant role in driving the inhibitor to adopt a suitable bioactive conformation oriented in the active site of enzyme. In general, this study is used as example to illustrate how multiple pharmacophore approach can be useful in identifying structurally diverse hits which may bind to all possible bioactive conformations available in the active site of enzyme. The strategy used in the current study could be appropriate to design drugs for other enzymes as well.

## Introduction

**Figure 1:**
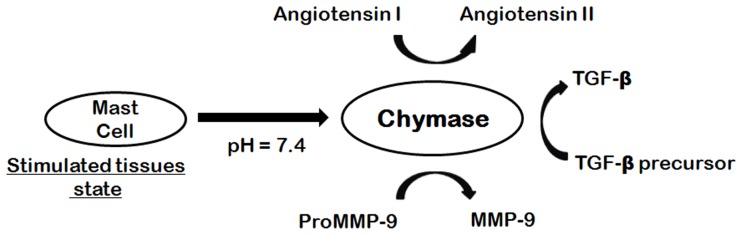
Chymase-dependent conversion of angiotensin I to angiotensin II and precursors of TGF-β and MMP-9 to their active forms.

Cardiovascular diseases are the leading cause of death in the developed world and are now on course to be emerging as the major cause of death in the developing world [1]. One particular manifestation of cardiovascular diseases, heart failure (HF), is dramatically increasing in frequency. A link between heart failure and chymase has been ascribed, and there is an interest to develop a specific chymase inhibitor as a new therapeutic regimen for the disease [2]. Chymase (EC 3.4.21.39) which is a chymotrypsin-like enzyme expressed in the secretory granule of mast cells, catalyzes the production of angiotensin I (Ang I) to angiotensin II (Ang II) in vascular tissues [3]. The octapeptide hormone, Ang II targets human heart and plays an important role in vascular proliferation, hypertension and atherosclerosis [4]. Conversion of Ang I to Ang II is also catalyzed by well-known angiotensin-converting enzyme (ACE), which is a metallo-proteinase with dipeptidyl-carboxypeptidase activity. However, chymase catalyzes the production of Ang II in vascular tissues even when ACE is blocked (Figure 1). Chymase converts Ang I to Ang II with greater efficiency and selectivity than ACE [5]. The rate of this conversion by chymase is approximately four fold higher than ACE. In order to generate Ang II, human chymase cleaves the Ang I at Phe8-His9 peptide bond. Chymase shows enzymatic activity immediately after its release into the interstitial tissues at pH 7.4 following various stimuli in tissues. Chymase also converts precursors of transforming growth factor-β (TGF-β) and matrix metalloproteinase (MMP)-9 to their active forms thus contributing to vascular response to injury (Figure 1). Both TGF-β and MMP-9 are involved in tissue inflammation and fibrosis, resulting in organ damage [6]. Previous studies have demonstrated the involvement of chymase in the escalation of dermatitis and chronic inflammation pursuing cardiac and pulmonary fibrosis [7]. Therefore, inhibition of chymase is likely to divulge therapeutic ways for the treatment of cardiovascular diseases, allergic inflammation, and fibrotic disorders. Chymase inhibition may also be useful for preventing the progression of type 2 diabetes, along with the prevention of diabetic retinopathy [8]. Moreover, role of chymase in inflammation has prompted its restorative value in diseases such as chronic obstructive pulmonary disease (COPD) and asthma [9].

Drug discovery and development is a time-consuming and costly procedure. Therefore, application and development of computational methods for lead generation and lead optimization in the drug discovery process are of immense importance in reducing the cycle time and cost as well as to amplify the productivity of drug discovery research [10]. These computational methods are generally categorized as ligand-based methods and (receptor) structure-based methods. In case of ligand-based methods, when biological activities of multiple hits are known, a more sophisticated class of computational techniques known as pharmacophore identification methods is often employed to deduce the essential features required for the biological activity [11]. A pharmacophore is an abstract description of molecular features which are necessary for molecular recognition of a ligand by a biological macromolecule. Due to the advantage in efficiency in the virtual screening, the pharmacophore model method is now a potent tool in the area of drug discovery [12]. However, the often cited drawback of the ligand-based methods is that they do not provide detailed structural information to help medicinal chemists in designing new molecules. The availability of the detailed structural information is critical especially during the lead optimization stage of the discovery process. While, structure-based pharmacophore methodology which involves generation of pharmacophore models directly from complex crystal structures is more reliable because it imposes the necessary constraints required for interaction and selectivity. Diverse inhibitor binding modes can be attained from ligand-based and structure-based pharmacophore modeling methodologies especially if many complex crystal structures are available for the target enzyme.

In this view, a strategy that integrates the advantages of multiple pharmacophore modeling and molecular docking approaches has been applied for the current study in order to identify compounds that contain the important chemical features to inhibit chymase enzyme. This strategy has been successfully applied for identification of compounds from the chemical database that can strongly bind at the active site of the target and thereby act as competitive inhibitors to the chymase. Finally, four druglike compounds from the database are reported as possible inhibitors for chymase enzyme. In final phase of current study, we have carried out herein Density Functional Theory-based quantum mechanical studies on potent hits retrieved by newly developed pharmacophore models. Various electronic properties such as LUMO, HOMO, and locations of molecular electrostatic potentials, are calculated for electronic features analysis. In general, the outcome of this research exertion demonstrates how multiple pharmacophore modeling accompanied with molecular docking, can be a significant approach in identification of hits compounds with high structurally diversity which may bind to all possible bioactive conformations available in the active site of enzyme. Moreover, this study is also expected to explore the molecular mechanism by which these compounds act and can be further utilized to get compounds with better activity by rational modification.

## Materials and Methods

### Receptor-ligand pharmacophore generation (structure-based approach)

**Figure 2:**
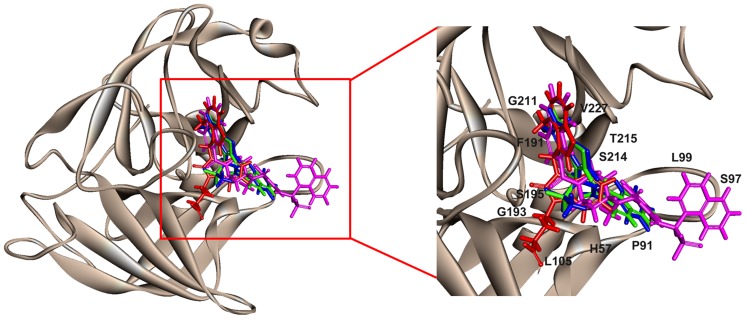
Crystal structure of chymase (PDBID: 3N7O). Available ligands that are co-crystallized with chymase were overlaid at the active site. These ligands with their bound conformations were used for Receptor-ligand pharmacophore generation. Zoomed view clearly shows the arrangement of residues at the active site.

Structure-based pharmacophore model utilizes the interactions between receptor-ligand complexes to generate a hypothesis [13]. As deposit of X-ray crystal structures in PDB is growing rapidly, the structure-based methods have become increasingly important. The information about the protein structure is a good source to bring forth the structure-based pharmacophore and used as first-screening before docking studies. To date, six crystal structures have been determined for human chymase as listed in Table 1. The four crystal structures which are co-crystallized with four different inhibitors include 3N7O, 1T31, 3SON, and 2HVX and their inhibitors are depicted in Figure 2. These crystal structures were downloaded from the Protein Data Bank (PDB). PDB is a repository for the 3-D structural data of large biological molecules, such as proteins and nucleic acids. The data, typically obtained by X-ray crystallography or NMR spectroscopy and submitted by biologists and biochemists from around the world, are freely accessible on the Internet via the website (http://www.rcsb.org). The PDB is overseen by an organization called the Worldwide Protein Data Bank, wwPDB. After downloading the desired crystal structures of chymase complexes, these four receptor-ligand complexes were used for development of structure-based pharmacophore models. The *Receptor-Ligand Pharmacophore Generation* protocol of Accelrys Discovery Studio v3.0 (DS), Accelrys, San Diego, USA, was applied to accomplish this task with default parameters. This protocol generates selective pharmacophore models based on receptor-ligand interactions. First, a set of features from the binding ligand is identified. The following predefined feature types are considered: hydrogen bond acceptor (HBA), hydrogen bond donor(HBD), hydrophobic(HY), negative ionizable(NI), positive ionizable(PI), ring aromatic(RA). Second, the pharmacophore models are ranked based on a measure of sensitivity and specificity and the top models are returned. The pharmacophore models are enumerated and then the selectivity is estimated based on a Genetic Function Approximation GFA) model. The GFA model for the selectivity of a pharmacophore is built from a training set of 3285 pharmacophore models. This set is used for searching the *CapDiverse* database in DS. The logarithmic values of the number of database search hits are used as the targets (a value of −1.0 is used if no hit is retrieved from the search). The number of total features in pharmacophore models and the feature-feature distance bin values are used as the descriptors for training the GFA model.

**Table 1:** List of Solved Crystal Structures of Human Chymase Co-Crystallized with Different Ligands.

PDB ID	Resolution (Å)	Ligand
3S0N	1.95	OBB
3N7O	1.80	N7O
2HVX	2.60	DRX
1T31	1.90	OHH
1PJP	2.20	_
1KLT	1.90	_

### Common feature pharmacophore generation (ligand-based approach)

**Figure 3:**
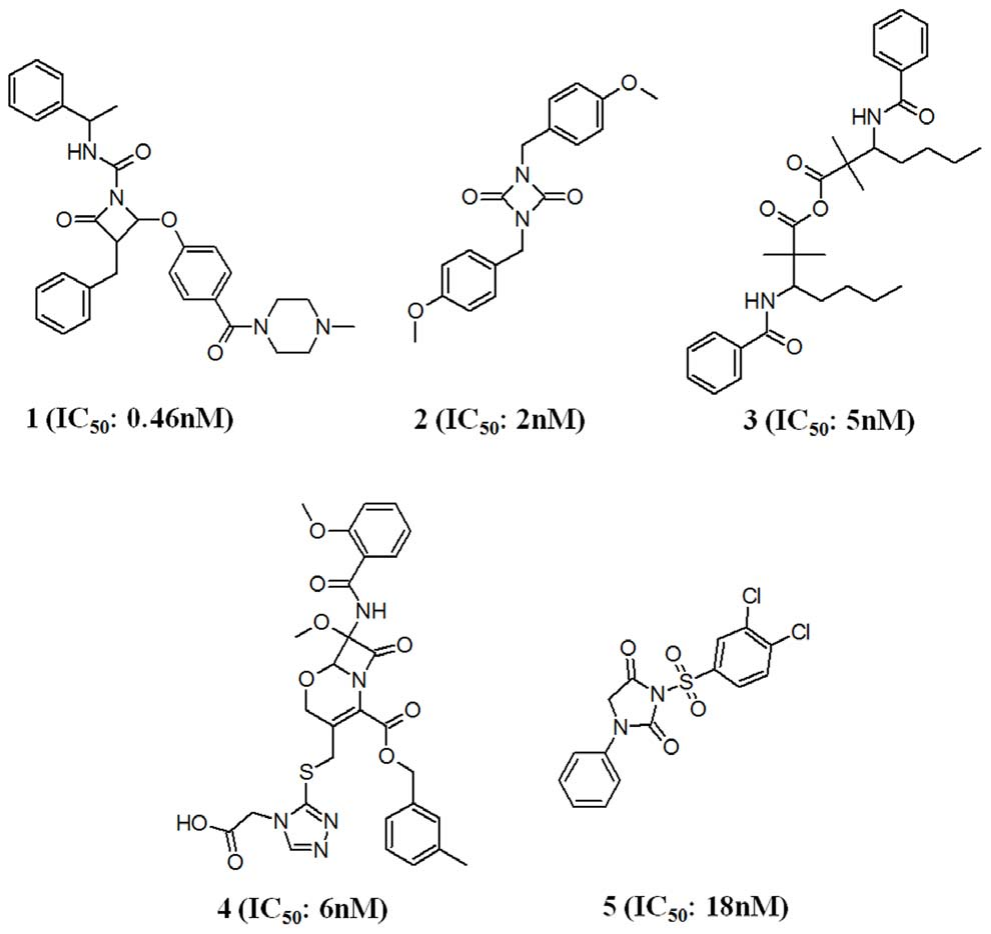
Training set compounds used in common feature pharmacophore generation.

Chemical compounds with their experimentally known chymase inhibitory activity(IC_50_) data were obtained from the literature such as life science journals, and a small database was compiled [14], [15], [16], [17], [18]. Chemical structures of these compounds were downloaded from BindingDB database (http://www.bindingdb.org). BindingDB is a public, web-accessible database of measured binding affinities, focusing chiefly on the interactions of protein considered to be drug-targets with small, drug-like molecules. BindingDB contains 947,406 binding data, for 6,667 protein targets and 393,164 small molecules. Five diverse compounds with the IC_50_ values less than or equal to 18 nM were selected as training set and employed in common feature pharmacophore generation calculation (Figure 3). A principal value of 2 and maximum omit feature value of 0 were assigned to the most active compound in the training set whereas 1 was assigned for the other compounds to label them as moderately active. For all compounds in the training set, energy minimization process was performed with *CHARMM* forcefield. Poling algorithm was applied to generate a maximum of 255 diverse conformations with the energy threshold of 20 kcal mol^−1^ above the calculated energy minimum for every compound in the dataset. These conformers were generated using *Diverse Conformer Generation* protocol running with *Best/Flexible* conformer generation option as available in DS. All five training set compounds associated with their conformations were used in common feature pharmacophore generation. *HipHop* module of the catalyst which was popularly known for Common Feature Pharmacophore Generation is available in DS as *Common Feature Pharmacophore Generation* protocol. *Feature Mapping* protocol was used to identify the common chemical groups present in the training set compounds. As predicted, hydrogen bond acceptor (HBA), hydrophobic aliphatic (HY_AL) and hydrophobic aromatic (HY_AR) features were selected during the pharmacophore generation.

### Validation of structure and ligand-based pharmacophore models

The purpose of the pharmacophore validation is to evaluate the quality of a pharmacophore model [11]. The capability to accurately predict internal and particularly external data sets is an important attribute of a reliable pharmacophore model [19]. The four structure-based models and best model from *Hip-Hop* module were validated using three different methods: (i) test set, to validate how well our selected pharmacophores pick the active from inactive compounds. In order to employ test set validation approach, a data set containing active and non-active compounds was prepared. Structurally diverse 134 compounds with a wide range of experimentally known chymase inhibitory activity values (2.1 to 40 000 nM) were merged with 190 presumably inactive compounds. This methodology of merging experimentally known active compounds with presumably inactive compounds has been successfully applied for validation of pharmacophore models in various studies [20], [21], [22]. Chemical structures of test set compounds were downloaded from BindingDB database (http://www.bindingdb.org). Thus, a test set containing 324 compounds was applied to determine the capability of the pharmacophore models to discriminate active compounds from other molecules in virtual screening process. (ii) The reliability of the generated pharmacophore models was also validated on the basis of the presence of chemical features essential to interact with the key amino acids in the active site of the corresponding target protein. (iii) Scale fit value method was also used to check the ability of pharmacophore models to differentiate between experimentally known chymase inhibitors based on their activity. For this purpose, a set of chymase inhibitors with a wide range of experimentally known chymase inhibitory activity was selected from literature. Chemical structures of these compounds were also downloaded from BindingDB database (http://www.bindingdb.org).

### Database preparation and multiple pharmacophore-based virtual screening

Maybridge (http://www.maybridge.com), and Chembrige (http://chembridge.com) which are commercial chemical databases containing 59 652, and 50 000 compounds, respectively, have been employed for virtual screening procedure. However, these databases are found to have number of nondruglike compounds. As, it is worthless to screen all the compounds of these databases and then eliminate them in the later phase for their nondruglike properties, therefore, the compounds not satisfying druglike properties were excluded from the databases prior to multiple pharmacophore-based virtual screening. In order to accomplish this task, compounds in these databases were subjected to various scrupulous druglike filters such as Lipinski's rule of five and ADMET (absorption, distribution, metabolism, excretion, and toxicity) properties. *Prepare Ligands* and *ADMET Descriptors* protocols as available in DS program were used in this step. After preparation of druglike databases, all structure-based and ligand-based pharmacophore models were subjected to screening of these druglike databases. The retrieved hits were further sorted out by applying filter such as maximum fit value of the best pharmacophore models from ligand-based and structure-based models, and were subsequently subjected to molecular docking process.

### Molecular docking

Molecular docking studies were carried out using GOLD (Genetic Optimization for Ligand Docking) 5.1 program from Cambridge Crystallographic Data Center, UK. GOLD uses a genetic algorithm for docking ligands into protein binding sites to explore the full range of ligand conformational flexibility with partial flexibility of protein [23]. Molecular docking was performed to generate the bioactive binding poses of inhibitors in the active site of enzyme. Protein coordinates from the crystal structure of chymase co-crystallized with N7O (PDB ID: 3N7O), determined at a resolution of 1.8Å were used to define the active site. All the water molecules present in the protein structure were removed and hydrogen atoms were added. The active site was defined with a 10 Å radius around the ligand present in the crystal structure. Ten docking runs were performed per structure unless three of the 10 poses were within 1.5 Å RMSD of each other. All the hit compounds as well as training set compounds were docked into chymase binding site. The GOLD fitness score is calculated from the contributions of hydrogen bond and van der Waals interactions between the protein and ligand, intramolecular hydrogen bonds and strains of the ligand. The interacting ability of a compound depends on the fitness score, greater the GOLD fitness score better the binding affinity. The protein – ligand interactions were examined by DS. Hit molecules which showed the expected interactions with the critical amino acids present in the active site of the protein, and comparable high binding scores than the bound ligand, were selected as potent inhibitors of chymase.

### Molecular docking validation using autodock 4.2

Autodock 4.2 was used to calculate the binding energies of the hit compounds at the active site of chymase [24]. The starting protein was prepared from the 1.8 Å resolution crystal structure of chymase labeled as 3N7O. We have chosen the highest resolution chymase crystal structure from the Protein Data Bank. Final hits along with training set compounds were docked using the Lamarckian genetic algorithm (LGA) in the “docking active site”, defined through a grid centered on the ligand of the complex structure. Population size of 150, mutation rate of 0.02, and crossover rate of 0.8 were set as the parameters. The default grid spacing (0.375 Å) was used. Simulations were performed using up to 2.5 million energy evaluations with a maximum of 27 000 generations. Each simulation was performed 10 times, yielding 10 docked conformations. The lowest energy conformations were regarded as the binding conformations between ligands and the protein.

### Validation of synthetic accessibility for hit compounds using SYLVIA

Synthetic accessibility scores for all four hit compounds were used to validate the synthetic possibilities. SYLVIA v 1.0 program from the Molecular Networks group was employed to calculate the synthetic accessibility of these optimized compounds [25]. The estimation of synthetic accessibility using SYLVIA provides a number between 1 and 10 for compounds that are very easy to synthesize and compounds that are very difficult to synthesize, respectively. The method for calculating synthetic accessibility takes account of a variety of criteria such as complexity of the molecular structure, complexity of the ring system, number of stereo centers, similarity to commercially available compounds, and potential for using powerful synthetic reactions. These criteria have been individually weighted to provide a single value for synthetic accessibility.

### Density Functional Theory (DFT) calculations

In the present study, we carried out a DFT-based quantitative structure–activity relationship (QSAR) study for both experimentally known chymase inhibitors and final hits. To obtain a significant correlation, it is fundamental that apposite descriptors be employed, whether they are theoretical, empirical, or experimental features of the structures. DFT is today one of the best methods to study medium size and larger molecular systems [26], [27]. The best DFT methods achieve substantially greater precision than the Hartree–Fock theory at only a modest augment in cost. They accomplish this task by incorporating few effects of electron correlation much less affluently than traditional correlated methods. A range of functional has been defined, generally distinguished by the manner that they treat the exchange and correlation components. The best known of the hybrid functionals is Becke's three-parameter formulation B3LYP [28]. Complete geometry optimization for data set compounds was carried out using DFT with B3LYP, using basis set 6-31G* level. A useful kind of net atomic charges, called electrostatic potential (ESP)-fitting charges, were derived from the DFT calculated molecular electrostatic potential distribution using CHelpG method, which produces charges fit to the electrostatic potential at points selected. Vibrational frequencies were computed at the same B3LYP/6-31G* level to characterize the stationary points on the corresponding potential energy surfaces. All calculations were performed using the Gaussian 09 suite of programs.

The experimentally known and highly active chymase inhibitors with substantial structural diversity which were used for the common feature pharmacophore generation were selected for DFT calculations. Moreover, four final hits KM09155, HTS00581, HTS0589, and Compound1192 retrieved from databases by the selected pharmacophore models, which showed important results with respect to all properties like key molecular interactions with the active site components, calculated drug-like properties, and high GOLD fitness score, were also designated for DFT study. Various quantum-chemical descriptors such as LUMO, HOMO, and locations of molecular electrostatic potentials (MESP) were computed.

### Calculation of molecular electrostatic potential (MESP)

For investigation of biologically active compounds, the mapping of the electrostatic potential is a well-known approach because it plays a key role in the initial steps of ligand-receptor interactions [29], [30], [31]. The formatted checkpoint files of the compounds generated by the geometric optimization computation were employed as input for *CUBEGEN* program interfaced with *Gaussian 09* program to compute the MESP. The MESP isopotential surface was produced and superimposed onto the total electron density surface (0.0004 e/au^3^). The electrostatic potential of the whole molecule was finally obtained by superimposing the electrostatic potentials upon the total electron density surface of the compound.

## Results and Discussion

### Generation of structure-based pharmacophore models

**Figure 4:**
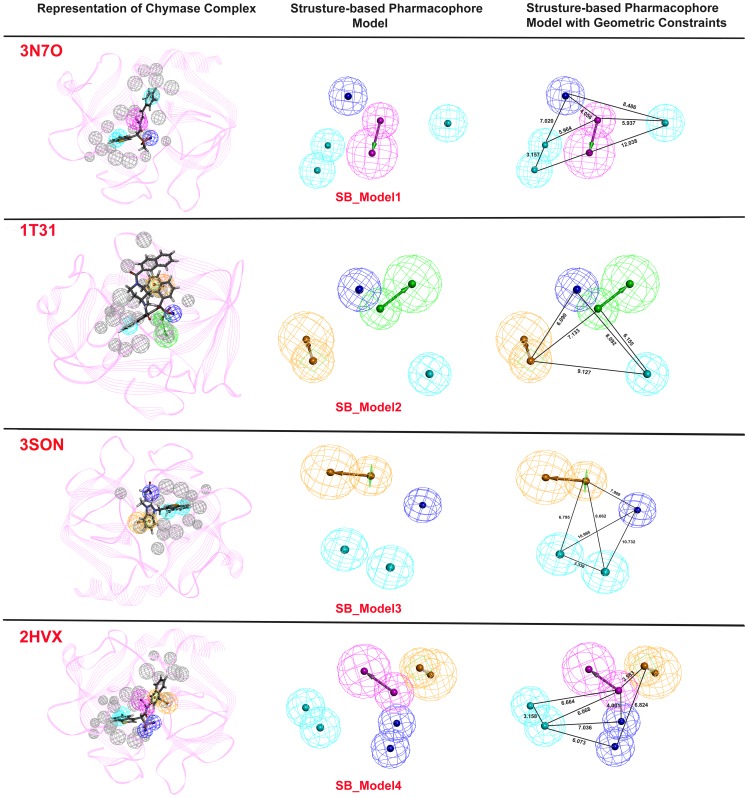
Representation of structure-based pharmacophore models with their geometric constraints. Cyan color shows hydrophobic (HY); magenta indicates hydrogen bond donor (HBD); green color indicates hydrogen bond acceptor (HBA); brown color denotes to ring aromatic (RA); and negative ionizable (NI) is shown in blue color.

The *Receptor-Ligand Pharmacophore Generation* protocol of DS presents the chemical features which instigate key interactions between protein and ligand as well as some excluded volume spheres corresponding to the 3D structure of protein. In this study, four different 3D structures of chymase bound with its inhibitors such as 3N7O, 1T31, 3SON, and 2HVX were selected as input for structure-based pharmacophore generation [9], [32], [33], [34]. The generated four pharmacophore models along with their excluded volume spheres and geometrical constrain are illustrated in Figure 4. The excluded volume spheres presented in our models provide an insight regarding the disallowed regions in the binding site. In general, these excluded volumes attempt to penalize molecules occupying steric regions that are not occupied by active molecules. Refinement of the pharmacophore with these excluded volume features provides a more selective model to reduce false positives and a better enrichment rate in virtual screening. In an attempt to account protein flexibility and reorganization effects at the pharmacophore level, the size of the excluded volume was set to 5Å to increase the effective size of the binding cavity. For 3N7O complex, the generated structure-based pharmacophore model (SB_ Model1) identified five functional features along with 20 excluded volume spheres, including one HBD pointed towards Ser214, one NI pointed to Lys40, and three HY centers pointed towards Tyr215, Gly216, and Leu99 amino acids, respectively. Pharmacophore model (SB_Model2) with four distinct features was generated from 1T31 complex. It composed of one HBA, one NI, one HY, and one RA with 20 excluded volume spheres. The HBA and NI features were directed to Gly193 and Lys192, respectively. While, RA feature of the SB_Model2 was pointed towards His57 amino acid of the active site of chymase. The pharmacophore model (SB_Model3) developed from 3SON complex also consists of four features with two HY features pointing in the direction of Gly199 and Arg200, one NI, and one RA pointing towards His45 along with 16 excluded volume spheres. The final pharmacophore model (SB_Model4) derived from 2HVX complex showed six features encompassing one HBD, two HY, two NI, and one RA with 23 excluded volume spheres. The two HY groups were pointed towards Phe191 and Gly216, and HBD pointed towards Tyr215. While, the RA feature was directed towards His57 and two NI features were pointed in the direction of Lys192 and Gly193. The comparison of above four pharmacophore models showed that hydrophobic feature was the common feature among all developed pharmacophore models. A previous study also showed that presence of hydrophobic sites for a chymase inhibitor were important for its effective binding with the key residues of the active site [35]. Pharmacophoric features of the models were directed towards key amino acids like Tyr215, His57, Lys192, Gly193, and Ser195 which play a major role in chymase inhibition activity [9], [34]. Hence, these features can be considered as important chemical features to discover the novel chymase inhibitors.

### Generation of ligand-based pharmacophore model

**Figure 5:**
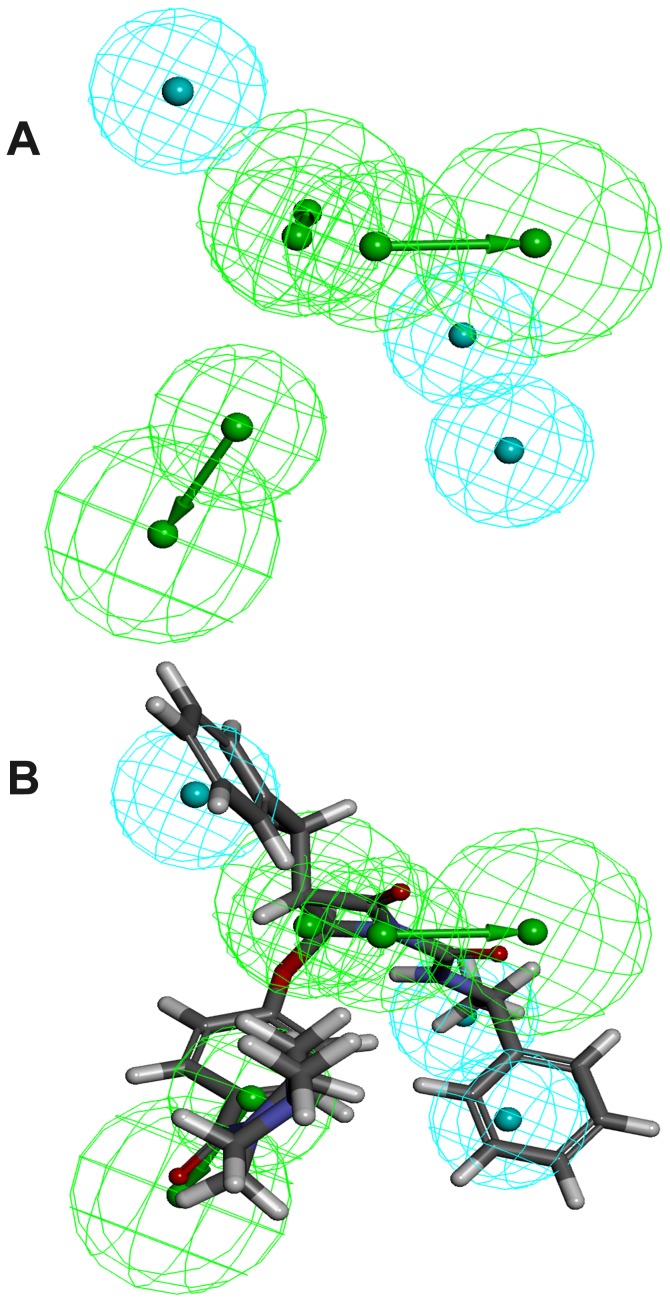
Ligand-based pharmacophore model (LB_Model) and its overlay on most active compound of training set.

Common feature pharmacophore models were generated for the target protein using set of experimentally known inhibitors. With the aim of acquiring a best model, numerous common feature pharmacophore generation runs were performed by altering the parameters such as minimum interfeature distance values, maximum omit feature, and the permutation of pharmacophoric features. The qualitative top ten pharmacophore models were developed (Table 2) using Common *Feature Pharmacophore Generation*/*DS* to identify the common features necessary to inhibit chymase. Direct and partial hit mask value of ‘1’ and ‘0’ for models connoted that the molecules present in dataset were well mapped to all the chemical features in the models and there is no partial mapping or missing features. The *Cluster analysis* was used to evaluate and categorize the difference between the compositions of models' chemical features and locations. These models could be roughly classified into two clusters according to the pharmacophoric features presented. The first eight models in cluster I identified six functional features, including three HBA, hydrophobic aromatic (HY_AR), hydrophobic aliphatic (HY_AL), and ring aromatic (RA) centers. The models in cluster II recognized five functional features, with two HBA, one HY_AL, and two RA. The distances between some pharmacophoric features in all models were rather constant, whereas some distances fluctuated in a relatively broad range, which indicated divergent tolerance of different features to spatial variation and provided rationale for further structural modification and optimization. As three models in cluster I showed higher ranking score and best fit values of the training set compounds, therefore these models were further evaluated to find the best model. There is not much difference in the ranking score among these models; therefore, an analysis of the best fit values of the training set compounds was carried out to choose the best model. The calculated best fit values designated Model 1 as the best and final ligand-based model (LB_Model) (Figure 5A). This final LB_Model which consists of three HBA, one HY_AL and two HY_AR features was further overlaid on the most active compound of training set (Figure 5B). The prevalence of HBA features in LB_Model derived from experimentally known inhibitors indicated that these chemical features were essential for the inhibition of chymase. A previous study also illustrated that HBA features in chymase inhibitors improve its binding affinity to the active site of chymase [36].

**Table 2:** Summary of the Pharmacophore Models Generated Using Hip-Hop for Chymase.

hypothesis	features	rank	direct hit	partial hit	max. fit
Hypo1	ZZHAAA	79.084	11111	00000	6
Hypo2	RZHAAA	78.571	11111	00000	6
Hypo3	RZHAAA	78.285	11111	00000	6
Hypo4	RRHAAA	77.768	11111	00000	6
Hypo5	RZHAAA	76.653	11111	00000	6
Hypo6	ZZHAAA	74.628	11111	00000	6
Hypo7	RZHAAA	74.583	11111	00000	6
Hypo8	RZHAAA	74.053	11111	00000	6
Hypo9	RRHAA	72.502	11111	00000	5
Hypo10	RRHAA	72.502	11111	00000	5

### Validation of structure and ligand-based pharmacophore models

A valid pharmacophore model should be not only statistically robust, but also predictive to internal and external data sets. Its capability to reliably predict external data sets and discriminate active inhibitors from other molecules is critical criteria for high-quality models. In this study, two validation methods are used to validate the quality of generated pharmacophore models which are as following.

### Test set method

**Figure 6:**
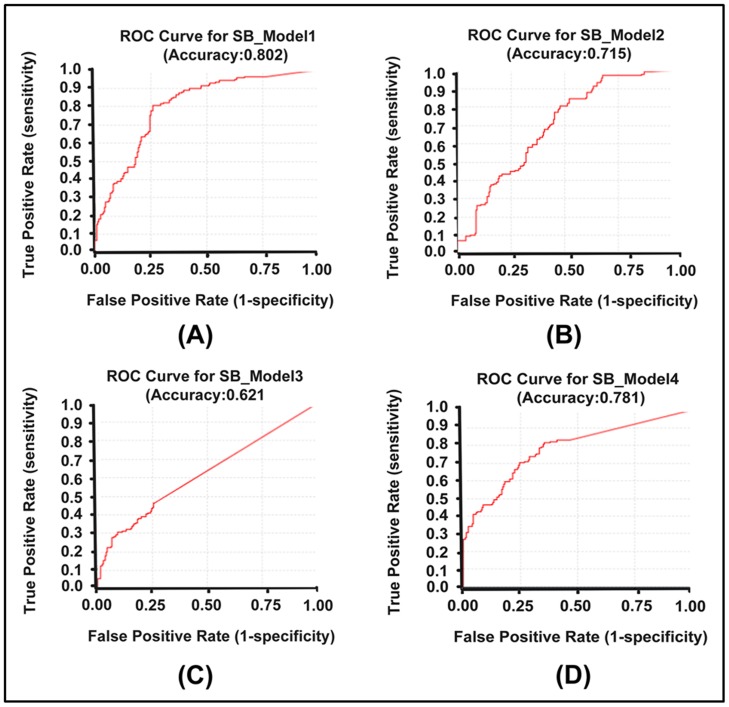
ROC curves generated for structure-based pharmacophore models by DS.

In order to perform test set validation technique which is considered as a meaningful approach to validate the discriminative power of a pharmacophore model in virtual screening, 134 compounds with a wide range of experimentally known chymase inhibitory activity values (2.1 to 40 000 nM) were used with 190 presumably inactive compounds. Thus, a test set containing 324 compounds was prepared for validation of pharmacophore models. All four structure-based pharmacophore models were validated using validation option of the *Receptor-Ligand Pharmacophore Generation* protocol of DS. By using this option of validation, both sensitivity and specificity of the models were calculated. Moreover, ROC curve was also generated for each structure-based pharmacophore model (Figure 6). SB_model1 with accuracy rate of 0.802, showed best predicted ability with high sensitivity and specificity. While, SB_mode3 with accuracy rate of 0.621 exhibited lowest predicted ability. The statistically significant parameters related to this validation technique are listed in Table 3 which clearly indicate that SB_Model1, SB_Model2, and SB_Model4 were able to distinguish between active and non-active compounds more precisely than SB_Model3. Therefore, these three models were selected for further evaluation. The ligand-based model (LB_Model) was also validated with the test set method. *Ligand Pharmacophore Mapping* protocol running with *BEST*/*Flexible* conformation generation option was used to map the test set compounds. LB_Model was able to predict 118 from total of 134 active compounds. Thus, it exhibited good sensitivity and specificity and was designated for further processing.

**Table 3:** Statistical Details of Test Set Validation Method Using DS.

Validation With Known Actives/Inactives								
Pharmacophore	Total Actives	Total Inactives	True Positives	True Negatives	False Positives	False Negatives	Sensitivity	Specificity
SB_Model1	134	190	129	47	143	5	0.96269	0.24737
SB_Model2	134	190	127	67	123	7	0.94776	0.33158
SB_Model3	134	190	62	141	49	72	0.46269	0.74211
SB_Model4	134	190	111	103	87	23	0.82836	0.54737
LB_Model	134	190	118	79	111	16	0.88060	0.41579

### Presence of chemical features essential to interact with key active site residues

**Figure 7:**
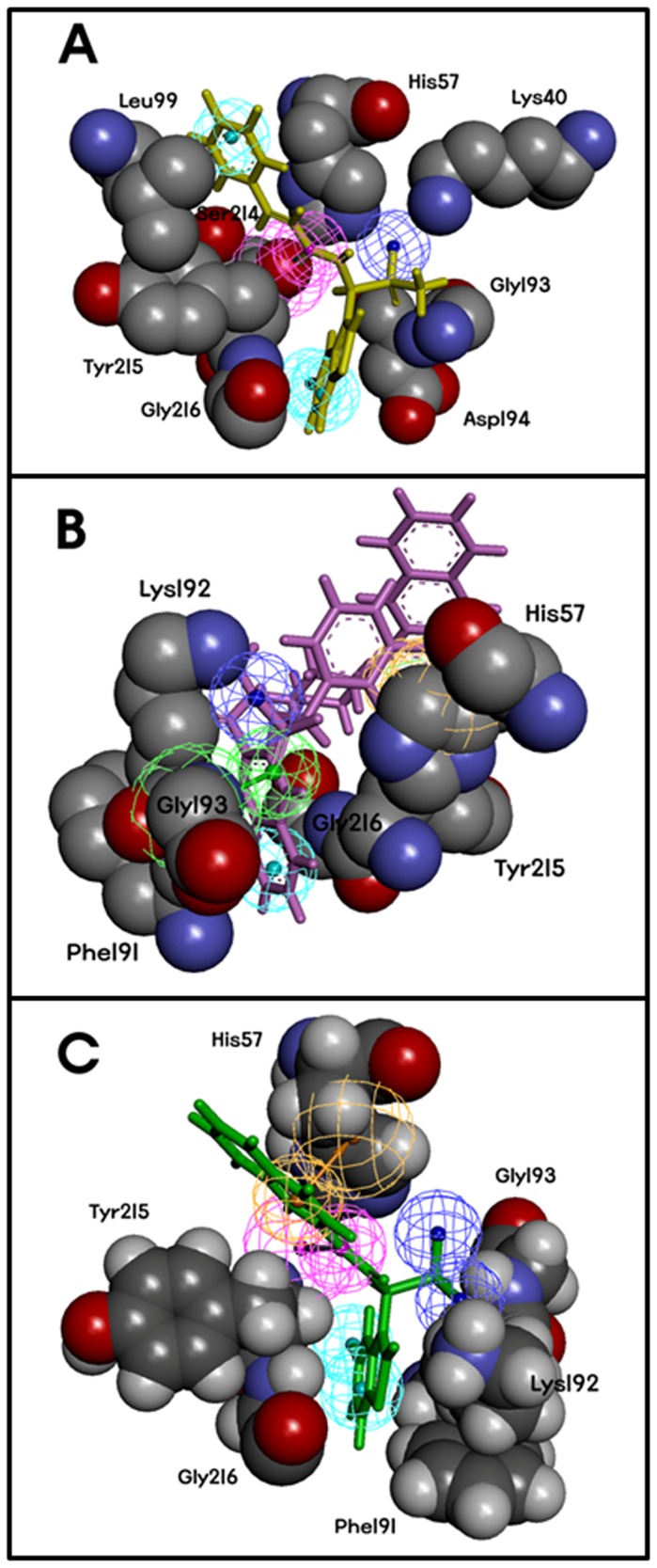
Overview of the interaction mode between chymase and known inhibitors at the active site of enzyme. (**A**) SB_Model1 mapped to N7O, (**B**) SB_Model2 mapped to OHH, (**C**) SB_Model4 mapped to DRX. Key amino acids involved in the interaction were displayed (gray: C atoms; red: O atoms; blue: N atoms).

**Figure 8:**
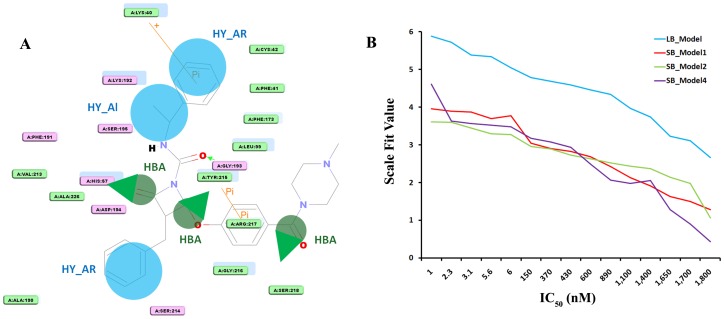
Ligand-protein interaction diagram from the chymase-inhibitor (compound 1 in the training set of chymase). (**A**). The pharmacophore mapping of the same compound is also depicted. HBA, hydrogen bond acceptor; HY_AR, hydrophobic aromatic, HY_Al, hydrophobic aliphatic. The locations of amino acid residues are represented in rectangular boxes, where pink and green colors denote both the hydrogen bond acceptor/donor and nonpolar contacts, respectively (**B**) Correlation graph between experimental IC_50_ and scale fit values.

Another method employed to validate the quality of all four phrmacophore models was the evaluation of models for the presence of chemical features required to interact with key active site residues. To find out the existence of chemical features that are complementary to the active site, diagrams were generated for the chymase-inhibitor complexes by using DS which illustrated the amino acids complemented to every feature present in the pharmacophore models (Figure 7). Overlay of the bound inhibitor on SB_Model1 connoted that chemical features of pharmacophore model were located in such a way to interact with important amino acids like Tyr215, Lys40, and Gly193. Chemical features of SB_Model2 were also oriented towards key amino acids like His57, Gly193 and Lys192. SB_Model4 also exhibited chemical features pointed to key residues of active site such as Lys192, Gly193, and Tyr215. In case of ligand-based pharmacophore model, the overlay of most active compound of the training set on LB_Model and docking of this compound into the active site of chymase clearly demonstrated that the three HBA, two HY_AR, and one HY_AL features of LB_Model have engendered numerous imperative interactions with key amino acids such as Lys40, His57, Lys192, Gly193, and Ser195 (Figure 8A). Thus, presence of chemical features essential to interact with key active site residues and discriminative power of developed models to active chymase inhibitors implicated that multiple pharmacophore-based virtual screening may provide an efficient approach to find novel chymase inhibitors from available databases.

### Scale fit value method

Third method to validate the generated ligand and structure-based pharmacophore models is the scale fit value method. The main purpose of this validation method is to verify the ability of pharmacophore models to distinguish between experimentally known chymase inhibitors based on their activity values. A set of 20 chymase inhibitors with diverse range of activity values from 1 nM to 1800 nM was selected and mapped over generated pharmacophore models. Results of this pharmacophore mapping over chymase inhibitors returned various fit values. A meticulous analysis of these fit values revealed that there was a good correlation between experimentally known activity values and fit values generated by pharmacophore mapping (Figure 8B). Thus, the result of this validation technique clearly indicates that the selected ligand and structure-based pharmacophore models have the capability to single out most active inhibitors form less active chymase inhibitors.

### Multiple pharmacophore-based virtual screening

To further validate representative pharmacophore models and demonstrate their efficiency, SB_Model1, SB_Model2, SB_Model4, and LB_Model were used as 3D queries to screen the chemical databases like Maybridge and Chembridge which consist of 59 652 and 50 000 compounds, respectively. Prior to multiple pharmacophore-based virtual screening experiments, both databases were transformed to druglike databases by *Prepare Ligands* and *ADMET Descriptors* protocols of DS. After preparation of druglike databases, all four pharmacophore models were subjected to screening of these druglike databases. For SB_Model4 which holds six features, Maximum omitted feature was set to 1 and for all other three models it was set to 0. The retrieved database hits were then ranked by their fit scores and the sorted list of hit compounds was analyzed to generate the final hits for each pharmacophore model. The hits acquired by the structure-based pharmacophore models with fit values above 2.0 were considered as potential hits and were reserved for further inspection. For LB_Model, fit value was set to3.5. The numbers of final hit compounds predicted by each of the four pharmacophore models from both databases are summarized in Table 4. It is observed that even for the same target, the hits retrieved by diverse pharmacophore models are quite distinguished from each other hence signifying that different pharmacophore models may show assorted output in virtual screening experiments. However, there were few common hits which were retrieved by more than one pharmacophore models. In order to decipher the proportion of common hits between various models, the overlap segment of the hit compounds obtained by each pair of two diverse pharmacophore models was evaluated. Analysis revealed that ratio of common hits among all four pharmacophore was between 18 and 32% thus showing the diversity in screening competency of different pharmacophore models derived from different complex structures of same enzyme. Consequently, multiple pharmacophore model-based screening approach should be applied to acquire better screening results. Finally, 133 hits compounds retrieved from database screening process were subjected to molecular docking studies.

**Table 4:** The Database Screening Results by Different Structure-based and Ligand-based Pharmacophore Models.

Pharmacophore Model	Hits Retrieved
SB_Model1	27
SB_Model2	47
SB_Model4	38
LB_Model	21

### Molecular docking

Docking experiments can be employed to answer various queries. For instance, position and orientation of an inhibitor or substrate can be predicted. An attempt to identify compounds that have affinity for the protein from a large database of compounds can be made. Moreover, prediction for any given molecule whether or not it has affinity for the protein, can also be done. Herein, we will present and discuss our docking experiments to address these issues for the chymase enzyme.

### Validation of ligand binding mode

**Figure 9:**
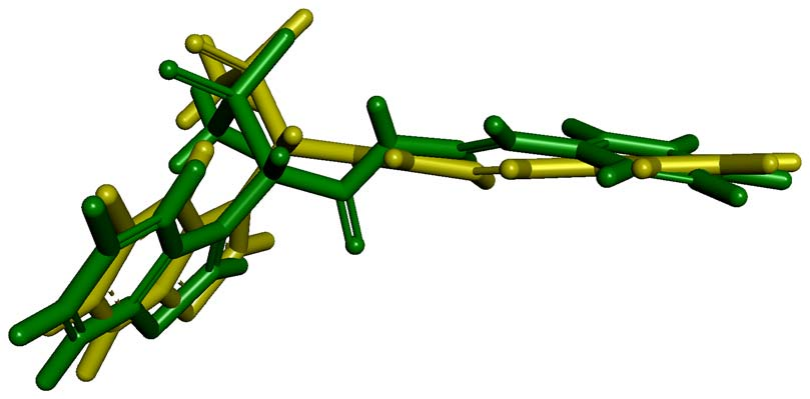
Overlay of the docked pose (green) of inhibitor with its crystal structure conformation (yellow).

Docking study has been performed with GOLD 5.1. An initial validation of the docking protocol is performed by comparing the conformation, position, and orientation (the pose) of a ligand as obtained from docking with the one determined experimentally with X-ray crystallography. Correctly redocking the crystallographically observed inhibitor is a minimum requirement to determine whether the program is applicable to this system or not. Crystal structure with the PDB code 3N7O bound with an inhibitor molecule (N7O) was selected as receptor and the active site was defined with a 10 Å radius around the ligand present in the crystal structure. The top conformation of ligand predicted by GOLD program was very close to the crystal structure-bound conformation. The RMSD between the docked pose and its bound conformation in the crystal structure is 0.53 Å indicating that GOLD is able to reproduce correct pose (Figure 9).

### Identification of hit compounds

After validation of the docking protocol, all 133 hits retrieved by employing a multiple pharmacophore model-based screening, were docked into the active site of chymase. Analysis of docking results indicated that bound ligand in the complex structure of chymase showed *GOLD* fitness score of 62.58. While, among 133 hit compounds, 21 hits demonstrated higher *GOLD* fitness score than the ligand bound with crystal structure, thus, were selected for further study. In order to obtain hits which could map all available bioactive conformations at the active site of chymase, these 21 hit compounds were further docked to the other two crystal structures of chymase labeled as 1T31 and 2HVX. These two crystal structures were also employed for the development of SB_Model2 and SB_Model4. Analysis of their docked results helped in further filtering of hit compounds. Finally, four hit compounds which showed the key interactions with the critical amino acids present in the active site of protein and also exhibited higher fitness score in all three crystal structures of chymase were selected as final hits. The final hits which included KM09155, HTS00581, and HTS05891 compounds, were retrieved from Maybridge database. While, fourth hit Compound1192 was retrieved from Chembridge database. Remarkably, all final hits were identified by four different pharmacophore models. KM09155 was revealed by LB_Model with fitness value of 4.36. Although, there were three compounds retrieved by LB_Model which showed high fitness scores than KM09155, however, could not show high fitness score for structure-based models. Therefore, these compounds were not selected as final hits. The HTS00581 hit was spotted by SB_Mode2 with fitness value of 3.83. While, the third hit compound HTS05891 was also marked by SB_Mode2 with 3.68 fitness score. The fourth final hit Compound1192 was identified by two different pharmacophore models including SB_Mode1 and SB_Mode4 with fitness scores of 3.50 and 3.72, respectively. Structural diversity of final hits was measured by using *Calculate Diversity Metrics* protocol of DS which calculates a series of quantitative measures of diversity including number fingerprint features, number assemblies, fingerprint distances, property distances and fraction cells. Result with *Diversity_NumAssemblies* value of 1.0 designated the final hits very high structural diversity. Therefore, it is quite evident that multiple pharmacophore-based virtual screening experiments merged with molecular docking studies are very competent tools for the identification of diverse hits in the drug discovery process.

### Validation of final hits using Autodock and SYLVIA

The binding modes of the potential chymase hits were further evaluated by using AutoDock 4.2 docking programs. The starting protein was prepared from the 1.8 Å resolution crystal structure of the 3N7O complex. Final four hits along with training set compounds were docked using the Lamarckian genetic algorithm (LGA) in the “docking active site”, defined through a grid (coordinates: X = −12.224, Y = −45.425, Z = −55.878). Although, AutoDock consumes more calculation time yet envisages the binding conformations more precisely [37]. It also computes torsional energy which gives rise to the binding energy of the docked compound. Autodock result signified that all the four hit compounds had scored similar or better binding energy values compared to the most active compound in the training set thus validating the output of GOLD docking program. In order to further validate final hit compounds, two more crystal structures of chymase deposited in protein data bank labeled as 1T31 and 2HVX were used for AutoDock validation. The resultant binding energies of hits with these structures also showed better or equal values compared to the binding energies of experimentally known potent chymase inhibitors present in the training set. To further validate our inhibition strategy, the synthetic accessibility of the final four hits was also measured using SYLVIA 1.0 program. The synthetic accessibility of most active compound **1** of the training set was also calculated for comparison purpose. The SYLVIA score of 6.19 for compound **1** was much high than the scores of final hits. Thus, the SYLVIA score for the final hits clearly illustrates that these compounds are easy to be synthesized (Table 5).

**Table 5:** GOLD Fitness Scores, AutoDock Binding Energies, and SYLVIA Synthetic Accessibility Scores of Potential Chymase Inhibitors Identified in This Study.

compound	Gold fitness (3N7O)	binding energy(3N7O)	Gold fitness (1T31)	binding energy(1T31)	Gold fitness (2HVX)	binding energy(2HVX)	SYLVIA Score
KM09155	77.533	−5.99	70.383	−5.68	73.365	−6.8	2.96
HTS00581	66.979	−4.66	66.915	−6.68	75.212	−7.73	4.68
HTS05891	64.781	−6.95	69.162	−7.06	67.096	−7.84	4.16
Comp.1192	63.872	−6.86	65.870	−7.55	64.419	−7.09	4.15

### Binding mode analysis of hits

**Figure 10:**
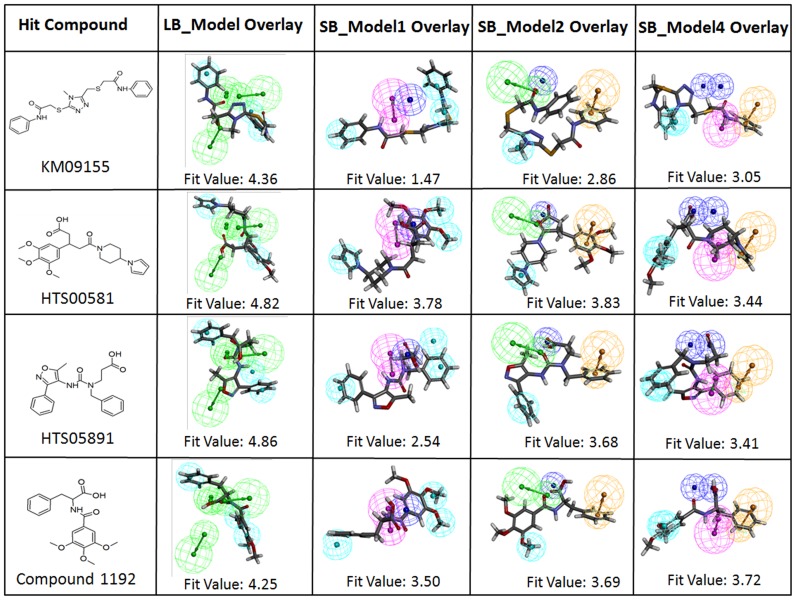
Identified hit compounds for chymase inhibition are overlaid on LB_Model, SB_Model1, SB_Model2, and SB_Model4, respectively.

**Figure 11:**
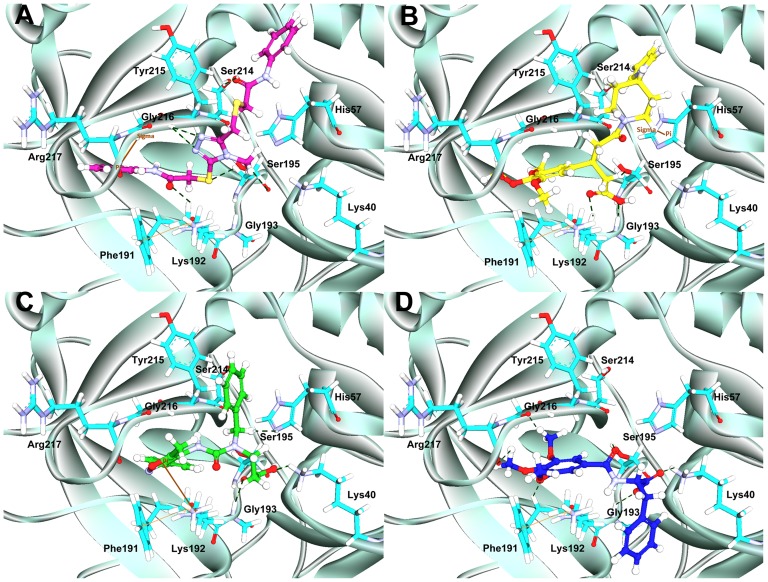
The molecular docking results. The binding modes and molecular interactions of hit compounds at the binding site of chymase enzyme: (**A**) KM09155, (**B**) HTS00581, (**C**) HTS05891 from Maybridge database, and (**D**) Compound1192 from Chembridge database. The key active site residues and inhibitors are shown in stick and ball-stick forms, respectively.

The 2D chemical structures of four hit compounds KM09155, HTS00581, HTS05891, and Compound1192 which were selected from the multiple pharmacophore-based screening and molecular docking studies, are illustrated in Figure 10. For all three crystal structures of chymase, the GOLD fitness score and the calculated binding energy values of final hits are given in Table 5. Moreover, the orientation and important interactions of the final hits with the key residues within the active site of chymase are shown in Figure 11. The analysis for binding mode of final hits within the active site region of enzyme is presented below.

### Binding mode of KM09155

This hit compound revealed very high GOLD fitness scores for all three crystal structures of chymase as compared to other three hits. KM09155 with maximum GOLD fitness score of 77.533 and minimum binding energy of −6.88 kcal/mol established a network of interactions with key amino acids like Ser195, Lys192, Ser214, and Gly216. The sulfur atom of the *N*-phenyl-2-sulfanylacetamide chemical moiety in KM09155 formed nonbonded electrostatic interaction with the carbonyl oxygen atom of Ser195. Carbonyl oxygen of *N*-phenyl-2-sulfanylacetamide also interacted with the nitrogen atom of Lys192. Moreover, imperative π^…^σ interactions between the phenyl ring system of KM09155 and the central carbon of Gly216 were also revealed. Important hydrogen bonded interactions were also elucidated between 4-methyl-4H-1,2,4-triazole ring of KM09155 and Ser195 and Gly216 amino acids. Although, KM09155 was revealed by LB_Model from database, it also mapped key features of other three structure-based pharmacophore models. Pharmacophoric overlay of this compound upon all four models is depicted in Figure 10.

### Binding mode of HTS00581

This compound was predicted with top GOLD fitness score of 75.212 and binding energy of −7.73 kcal/mol. At the active site of chymase, this compound has established various important close contacts with key amino acids. For instance, carbonyl oxygen atom of acetic acid group in HTS00581 hit compound fashioned hydrogen bond interaction with oxyanion hole formed by Ser195 and Gly193 amino acids. The π^…^σ interactions between the side chain imidazole ring of His57 and piperidine moiety of hit compound were also observed. This compound has gained substantial hydrophobic interactions at the active site region of enzyme. The SB_Model2 with four distinct chemical features including one HBA, one NI, one HY, and one RA retrieved HTS00581 hit compound from database with fitness score of 3.83. The mapping of HTS00581 hit over SB_Model2 is illustrated in Figure 10. Furthermore, along with the mapping of SB_Model1 and SB_Model4, the HTS00581 compound was also mapped over LB-Model in order to find out, whether this hit compound possesses the very basic chemical features which are present in currently available chymase inhibitors. It mapped all the features of LB-Model thus indicating the complimentary features that facilitate strong enzyme-ligand binding interactions.

### Binding mode of HTS05891

The binding mode of this hit compound at the active site of chymase has illustrated various kinds of interactions such as hydrogen bonding, π^…^cationic, and hydrophobic interactions with key residues in the active site region. An important π^…^cationic interaction between the 5-methylisoxazole ring of hit compound and nitrogen atom of Lys192 was elucidated. The presence of this electrostatic interaction lead to the binding orientation of hit compound in more favorable way which instigated key interactions with other key residues like Gly193 and Ser195. The oxygen atom of carbonyl group in hit compound formed very close hydrogen bond interaction to hydrogen atom of Gly193. Another key hydrogen bonding interaction was also observed between HTS05891 hit and Ser195 amino acid. Considerable hydrophobic interactions were also observed between the hit compound and active site of chymase. The HTS05891 hit was spotted by SB_Model2 from database and the overlay of pharmacophoric features of SB_Model2 on the compound is depicted in Figure 10. Moreover, this hit compound mapped all the features of LB-Model which contains essential chemical features present in experimentally known potent chymase inhibitors. It also mapped five features of the Sb_Model3. HTS05891 exhibited high GOLD fitness scores for all three crystal structures of chymase used in this docking study. The maximum GOLD fitness score of this compound for chymase binding is 67.096 with the binding energy of −7.84 kcal/mol.

### Binding mode of compound 1192

With maximum Gold fitness score of 65.870 and binding energy of −7.55 kcal/mol, this hit compound has established numerous close contacts that lead to the important ligand-enzyme interaction such as hydrogen bonding interactions with Gly193, Ser195, Phe191 and hydrophobic interactions with Gly216 and Arg217 amino acids in the active site of the enzyme (Figure 11). The oxygen atom of formic acid group present in compound1192 has shown close hydrogen bonding interaction with the nitrogen atom of Gly193. Hydrogen bonding was also observed between carbonyl group and Ser195 amino acid which is an important residue in the active site of chymase enzyme. Additional hydrogen bonding interactions were also elucidated between Phe191 and Arg217. Compound1192 was marked from database by two pharmacophore models including SB_Model1 and SB_Model4. This hit compound also mapped all the pharmacophoric features of SB_Model2. However, it missed one of the HBA features of LB_Model and mapped five features out of six for this pharmacophore model. As, compound1192 has mapped well four structure-based and ligand-based pharmacophore models, therefore, it indicates the presence of features imperative for strong enzyme-ligand binding interactions. The overlay of all four models on hit compound1192 is depicted in Figure 10.

### Density Functional Theory (DFT) Calculations

#### Electronic attributes

**Figure 12:**
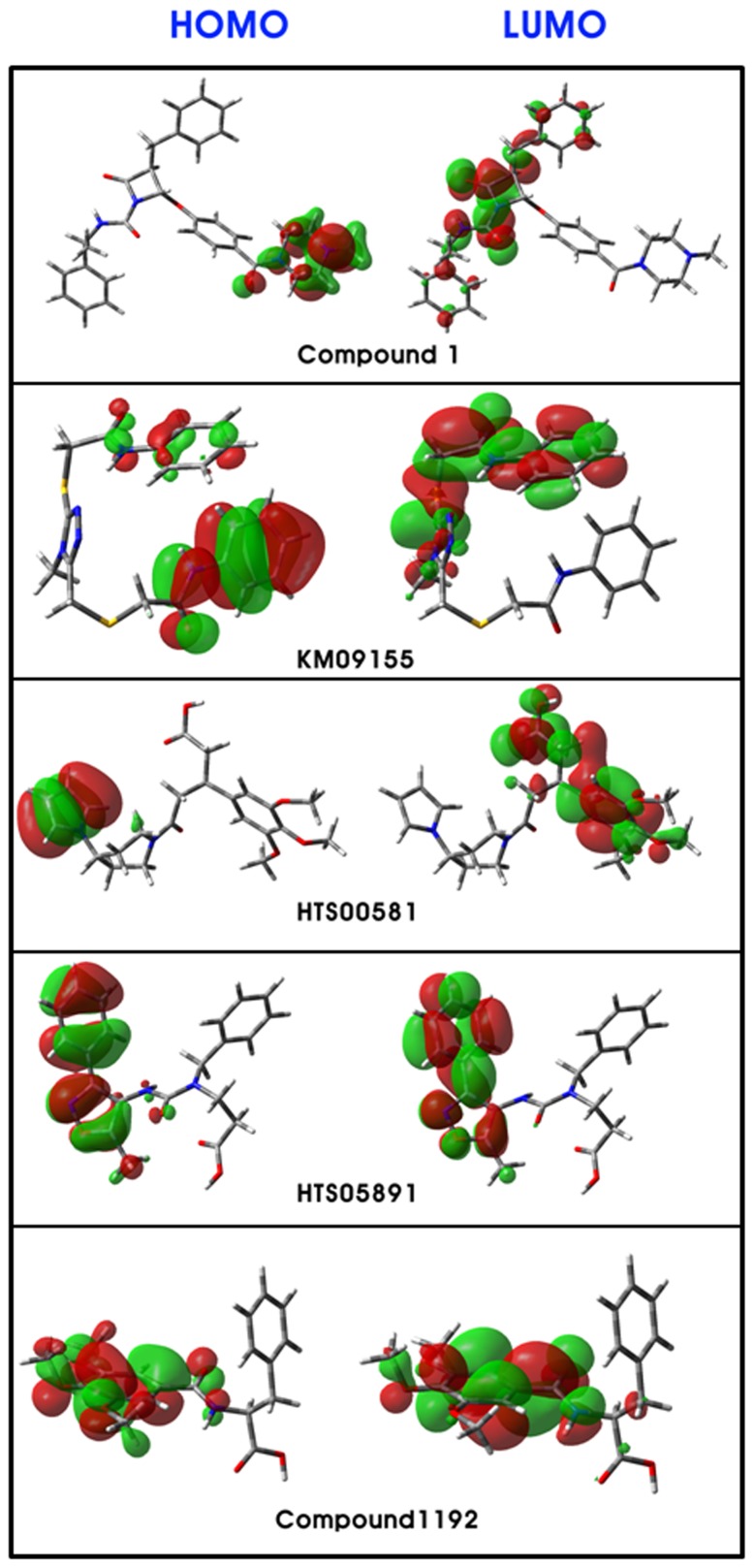
Plots of HOMO and LUMO of most active compound 1 along with potent hits KM09155, HTS00581, HTS05891 and Compound1192.

According to Frontier Orbital Theory, the shapes and symmetries of the HOMO and LUMO are crucial in predicting the reactivity of a species and the stereochemical and regiochemical outcome of a chemical reaction. Consequently, the outcome of these quantum chemical descriptors, direct us to distinguish the reactive sites and substituent influence on electronic structure of the compounds. Maps of HOMO and LUMO are plotted onto the molecular surfaces of all four hit compounds along with most active compound 1 of the training set (Figure 12). Inspection of variations in these maps of molecular orbitals indicates that electron exchange and electron-transfer ability of the compounds may have a role in their anti-chymase activity. The HOMO map delineates the area that is most electron-sufficient. Analysis of HOMO maps of compounds illustrate that HOMO molecular orbitals are located on aromatic and the heteroaromatic rings which contain the heteroatoms such as nitrogen and oxygen. While, inspection of LUMO plots demarcated the regions that can act as electron acceptors to the active site of the chymase. Amide groups and heteroaromatic rings were the most often groups in hit compounds occupied by LUMO orbitals. These results are quite consistent with the docking analysis which illustrates the participation of these moieties in the key ligand-receptor interactions. A previous experimental study also inferred that introduction of heteroatoms to the inhibitor compound enhanced its stability in human plasma [18]. For instance, the placement of an ethoxy group in compound 2 instigated its stability. Thus, the analysis of two frontier orbitals clearly indicates an important role of charge-transfer interactions with the binding site in the receptor for potent activity. Electron donating or withdrawing groups in the compounds may be responsible for an increase or decrease in the orbital energies by allowing modulation of the molecular electronic “band gaps”.

### Molecular electrostatic potential (MESP) profiles

**Figure 13:**
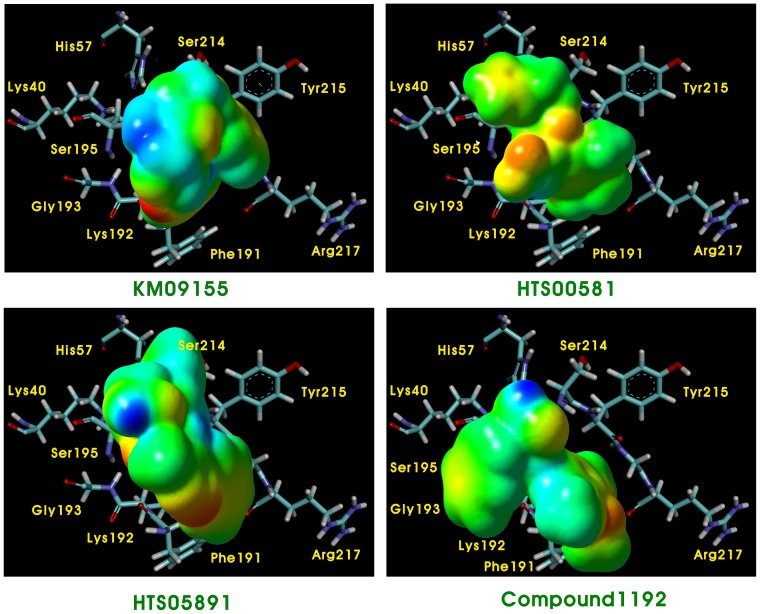
Differential maps of MESP for final hit compounds KM09155, HTS00581, HTS05891 and Compound1192. The red and the blue color represent the electronegative and electropositive potentials whereas the green represents a potential halfway between the two extremes.

Electrostatic potential characteristics are considered to be key features of molecules through which it recognizes its receptor. The molecular electrostatic potential surface MESP which is a plot of electrostatic potential mapped onto the iso-electron density surface, simultaneously displays molecular shape, size and electrostatic potential values. MESP mapping is very useful in the investigation of the molecular structure with its physiochemical property relationships. Nam et al. reported their discovery that electrostatic interactions accounted for the majority of the rate acceleration in the mechanism of RNA transphosphorylation in solution catalyzed by the hairpin ribozyme [38]. Moreover, the electrostatic funnel illuminated from three-dimensional mapping of the electrostatic potential was reported by Dehez et al., driving the diphosphate nucleotide rapidly toward the bottom of the internal cavity of membrane-protein mitochondrial ADP/ATP carrier by forming a privileged passageway [39]. Taking into account these findings comprehensively, we assumed that the electrostatic potential of the inhibitor also played a important role in the binding and interaction with chymase together with orbital energy and consequently influenced the inhibition effect. The 3D MESP plots of hit compounds were superimposed inside the active site of chymase (Figure 13). The coloring area of the surface represents the overall molecular charge distribution with the electrostatic potential. As for the compounds in this study, the electronegative potential (MESP_min_) was coded with red on the MESP maps while the interpolated blue map represents the electropositive potential (MESP_max_) of a strongest repulsion. The predominance of green region in the MESP surfaces corresponds to a potential halfway between the two extremes that are indicated in red and blue colors, respectively. The MESP plotted onto constant electron density surface for KM09155 hit showed the most electropositive potential region at the methyl of 4-methyl-4H-1,2,4-triazole ring and the most electronegative potential region was spread over the oxygen atoms of the both carbonyl groups present in KM09155. In other hit compounds, hydrogen atoms attached with heteroatom like oxygen and nitrogen are the regions which bear the maximum brunt of positive charge. Moreover, a gradual depletion of both red and blue areas and an increase of green color around the aromatic rings were also observed. On the whole, appearance of both most electronegative and electropositive regions along with moderate section in hit compounds demonstrates that these regions can act as electron donors or acceptors to the active site of the chymase thus making these compounds very reactive. Docking results of these compounds also signified the participation of these areas in the imperative interactions with the key active site residues such as Ser195, Gly193, His57, Tyr215 and Phe191 of the enzyme.

## Conclusions

A deriving pharmacophore model from the three-dimensional structure of a target protein provides helpful information for analyzing protein-ligand interactions and further improvement of ligand binding affinity. While, pharmacophore model derived from already known inhibitors facilitates in the identification of essential chemical features present in experimentally known potent chymase inhibitors. To find novel and potent chymase inhibitors and to provide a new idea for drug design, we used both ligand-based and structure-based methods to perform the virtual screening (VS) of commercially available databases. As different pharmacophore models generated from different crystal structures may represent different inhibitor binding modes. Therefore, multiple pharmacophore-based virtual screening approach can be more efficient way in identification of potent hits that can bind to various bioactive conformations available in the active site of enzyme. X-ray crystallographic data of chymase in complex with different inhibitors were used to generate four structure–based pharmacophore models. A common feature pharmacophore model was also developed from experimentally known inhibitors. After successful validation of developed pharmacophore models, a smart virtual screening strategy was conducted by employing all pharmacophore models to retrieve hits with novel chemical scaffolds. Drug-like hit compounds were subjected to molecular docking using GOLD and AutoDock to evaluate compounds for important binding site interactions and affinity. Finally four structurally diverse compounds with high GOLD score and binding affinity for several crystal structures of chymase were selected as final hits. Identification of final hits by four different pharmacophore models necessitates the use of multiple pharmacophore-based approach in VS process. Quantum mechanical (QM) calculation is also conducted for analysis of electrostatic characteristics of compounds. Inspection of the molecular electrostatic potential surfaces and frontier molecular orbitals successfully explained their significant role in driving the inhibitor to adopt a suitable bioactive conformation oriented in the active site of enzyme. In general, this study is used as example to illustrate how multiple pharmacophore approach can be useful in identifying structurally diverse hits which may bind to all possible bioactive conformations available in the active site of enzyme. The present study may lead to the knowledge of chemical properties which are likely to improve activity of already known chymase inhibitors and may also allow the modification of the structure of new chemical entities (drug) for the improved bioavailability. The application of multiple pharmacophore-based VS can also be extended to the development of fast-follower drugs, where more than one high-quality crystal structures of the target in complex with potent ligands are already available. Therefore, the multiple pharmacophore modeling approach can be very useful in virtual screening of any chemical database for the development of new potent inhibitors for the enzyme.
